# *TNFRSF1A* Gene Polymorphism (−610 T > G, rs4149570) as a Predictor of Malnutrition and a Prognostic Factor in Patients Subjected to Intensity-Modulated Radiation Therapy Due to Head and Neck Cancer

**DOI:** 10.3390/cancers14143407

**Published:** 2022-07-13

**Authors:** Iwona Homa-Mlak, Radosław Mlak, Marcin Mazurek, Anna Brzozowska, Tomasz Powrózek, Mansur Rahnama-Hezavah, Teresa Małecka-Massalska

**Affiliations:** 1 Department of Human Physiology, Medical University of Lublin, Radziwiłłowska 11 St., 20-059 Lublin, Poland; radoslawmlak@gmail.com (R.M.); marcin.mazurek1@umlub.pl (M.M.); tomaszpowrozek@gmail.com (T.P.); teresamaleckamassalska@umlub.pl (T.M.-M.); 2 II Department of Radiotherapy, Center of Oncology of the Lublin Region St. John of Dukla, Jaczewskiego 7 St., 20-059 Lublin, Poland; annabrzo@poczta.onet.pl; 3 Chair and Department of Dental Surgery, Medical University of Lublin, 20-093 Lublin, Poland; mansur.rahnama@umlub.pl

**Keywords:** head and neck cancer, malnutrition, critical weight loss, nutritional deficiencies, polymorphism, *TNFRSF1A*

## Abstract

**Simple Summary:**

Malnutrition is frequently related to the increased level of proinflammatory cytokines. Tumor necrosis factor α is a proinflammatory cytokine that plays a pivotal role in the development of malnutrition and cachexia in cancer patients. This study aimed to assess the relationship between a functional polymorphism of the *TNFRSF1A* gene and the occurrence of nutritional disorders in patients subjected to radiotherapy due to head and neck cancer. Multivariable analysis revealed that the TT genotype of the *TNFRSF1A* gene (−610 T > G) was independently correlated with a higher risk of nutritional disorders. Determination of this polymorphism may be useful in the assessment of the risk of nutritional deficiencies in patients subjected to radiotherapy due to head and neck cancer.

**Abstract:**

*Background*: Malnutrition is a nutritional disorder observed in 52% of patients with head and neck cancer (HNC). Malnutrition is frequently related to the increased level of proinflammatory cytokines. In turn, ongoing inflammation is associated with increased catabolism of skeletal muscle and lipolysis. Tumor necrosis factor α (TNF-α) is a proinflammatory cytokine that plays a pivotal role in the development of malnutrition and cachexia in cancer patients. The aim of the study was to assess the relationship between a functional single-nucleotide polymorphism (SNP) −610 T > G (rs4149570) of the *TNFRSF1A* gene and the occurrence of nutritional disorders in patients subjected to RT due to HNC. *Methods*: The study group consisted of 77 patients with HNC treated at the Oncology Department of the Medical University in Lublin. Genotyping of the *TNFRSF1A* gene was performed using capillary electrophoresis (Genetic Analyzer 3500). *Results*: Multivariable analysis revealed that the TT genotype of the *TNFRSF1A* gene (−610 T > G) was an independent predictor of severe malnutrition (odds ratio—OR = 5.05; *p* = 0.0350). Moreover, the TT genotype of this gene was independently related to a higher risk of critical weight loss (CWL) (OR = 24.85; *p* = 0.0009). *Conclusions*: SNP (−610 T > G) of the *TNFRSF1A* may be a useful marker in the assessment of the risk of nutritional deficiencies in HNC patients treated with intensity-modulated radiotherapy (IMRT).

## 1. Introduction

In 2017, approximately 890,000 new cases of head and neck cancer (HNC) were diagnosed, which accounted for 5.3% of all cancers [1]. Despite the wide range of available therapeutic options, the results of treatment, especially in advanced stages, remain unsatisfactory [2]. Moreover, patients with HNC are second (after patients with gastrointestinal tract cancer and before lung cancer patients) most commonly burdened with nutritional deficiencies [3].

According to the Global Leadership Initiative on Malnutrition (GLIM), both phenotypic and etiologic criteria must be fulfilled to diagnose malnutrition. Two out of the three following phenotypic criteria must be met: nonvolitional weight loss, reduced body mass index (BMI), and reduced fat-free mass index (FFMI). The etiological criteria assume the occurrence of reduced food intake or absorption and disease burden which may or may not be accompanied by inflammatory conditions [4]. The most common type of neoplasm in HNC cases (90%) is squamous cell carcinoma (SCCHN) [5]. At diagnosis, malnutrition is observed even in even 52% of SCCHN patients [6]. After the implementation of treatment, the percentage of patients with malnutrition can increase to even 88% [6]. The possible causes of the dramatic increase in the occurrence of malnutrition during treatment include such side-effects as xerostomia, dysphagia, or oral mucositis (OM). Another major problem is tumor location. Tumors in the head and neck area can cause odynophagia, mechanical obstruction, dysphagia, and anorexia [6,7]. The occurrence of cancer malnutrition is associated with a higher rate of treatment (CTH and/or RT) toxicity, decreased quality of life (QoL), and poor prognosis [6,7,8]. DRM is usually accompanied by excessive release of systemic proinflammatory mediators (e.g., interleukin 1—IL-1, interleukin-6—IL-6, and tumor necrosis factor alpha—TNF-α). Interestingly, they can be produced in significant amounts not only by the host cells, but also by cancer cells [9]. The etiopathomechanism of malnutrition, despite the recognition of certain factors involved in its development (e.g., inflammation, hormonal changes causing appetite disorders, and catabolic changes in muscle and fat tissue) is still not fully understood [10]. TNF-α regulates various pathways of the immune system’s response, including the innate and cellular immune responses. In addition, TNF-α is produced by cancer cells and could be one of the main factors causing muscle (especially skeletal muscle) wasting in malnutrition [11].

TNF-α activates signals for pathways starting within two receptors: tumor necrosis factor receptor superfamily member 1A (TNFRSF1A (CD120a) and TNFRSF1B (CD120b) [12,13,14]. It plays a critical role in non-lysosomal and lysosomal proteolytic pathways, induction of the ubiquitin-proteasome pathway, decrease in protein synthesis, decrease in adipose tissue enzymes (e.g., lipoprotein-lipase), and simultaneous increase in adipose tissue lipolysis [7]. Some alterations of TNF receptor (TNFR1) function and signaling can result in inflammatory deregulation and, consequently, lead to the development of numerous diseases and disorders (e.g., malnutrition) [9,15]. The significant role of the systemic inflammatory response mediated by the activation of TNFR1 in the etiopathology of cancer-related malnutrition (CRM) encourages the investigation of the single-nucleotide polymorphisms (SNPs) of the *TNFRSF1A* gene as risk factors for nutritional deficiencies [10]. According to studies, SNP rs4149570 of the TNFRSF1A has functional consequences (it is related to altered mRNA level of this gene). To date this SNP has been studied in several non-neoplastic diseases, including inflammatory bowel disease and ankylosing spondylitis [16,17]. Moreover, the above SNP was also studied in four cancer types (HNC, lung cancer, colon cancer, and rectal cancer). In our previous study on patients treated for HNC using radiotherapy RT, we indicated an association between this SNP and the risk of more severe, therapy-induced oral mucositis [18].

We postulate that the SNP of *TNFRSF1A* (rs4149570) might be associated with a higher risk of the occurrence of nutritional deficiencies. Identification of risk factors of malnutrition may facilitate the development of prevention strategies or at least minimize its consequences [16,19]. However, to date, the abovementioned SNP has not been studied as a nutritional disorder risk factor or a prognostic factor; thus, our study seems to be warranted.

Therefore, the aim of this study was to assess *TNFRSF1A* gene polymorphism (−610 > G, rs4149570) as a predictor of malnutrition or prognostic factor in patients subjected to intensity-modulated radiotherapy (IMRT) due to HNC.

## 2. Material and Methods

### 2.1. Study Group

The study group consisted of 77 patients subjected to IMRT due to HNC. Blood samples were obtained from all patients. Sequencing and determination of *TNFRSF1A* SNP were performed in all 77 samples. This observational study was performed in a group of consecutively recruited patients who met inclusion and exclusion criteria. Inclusion criteria were as follows: age over 18 years, HNC confirmed by a histopathological examination, advanced cancer (stages III or IV), and use of IMRT as an element of the multimodal treatment. Exclusion criteria were as follows: autoimmune disease, active infection, and any coexisting or previous cancer. Excessive alcohol consumption (F10.1 and F10.2) was assessed according to the criteria of the International Statistical Classification of Diseases and Related Health Problems (ICD). Smoking status was defined as described below. An adult person who has smoked 100 cigarettes in their lifetime and who currently smokes was described as a current smoker. A person described above who had quit smoking by the time of the interview was defined as a former smoker.

The study was approved by the Bioethics Commission at the Medical University in Lublin (no KE-0254/232/2014). All patients in the study signed the informed consent. Patients were treated between 2014 and 2017 at the Oncology Department of the Medical University in Lublin located in St. John of Dukla Lublin Region Cancer Center.

### 2.2. Treatment

All patients were treated using IMRT (ONCOR linear accelerator, Siemens). Patients received 54–70 Gy (with a daily dose of 2 Gy). Patients in more advanced stages received a total dose of 70 Gy in 35 fractions onto the tumor and enlarged lymph nodes (LNs). Postsurgical patients with volume risk received a total dose of 66 Gy in 33 fractions. Patients with average or low volume risk received total doses of 60 and 54 Gy, respectively. Doses of 54 or 60 Gy were used in the elective treatment of LNs. In patients subjected to C-RT, cisplatin and 5-fluorouracil (FU) (1–4 cycles of chemotherapy—CTH) were used in addition to RT. The PF scheme administered in neoadjuvant CTH included cisplatin (100 mg/m^2^ on day 1) and 5-FU (1000 mg/m^2^ per day, continuous infusion on days 1–5) in 21-day cycles. In the course of concurrent chemoradiation, cisplatin was administered in the dose of 100 mg/m^2^ every 21 days.

### 2.3. Assessment of Disease Stage of Advancement, Patient’s Performance and Nutritional Status

The eighth edition of TNM classification was used in the assessment of disease stage of advancement. The patient performance status was evaluated according to the Eastern Cooperative Oncology Group (ECOG) scale. When statistical analysis required dichotomization of performance status (PS) data, the following categories: ≤1 or >1 were used. The medical examination included measurement of the patient’s height and weight, which were subsequently used for BMI calculation. Weight measurement and BMI calculations were performed three times: before, during (fourth week), and after (seventh week) RT (weight I and BMI I, weight II and BMI II, and weight III and BMI III, respectively).

The nutritional status of patients was assessed before the start of IMRT by means of the Nutritional Risk Score-2002 (NRS-2002) and Subjective Global Assessment (SGA). According to NRS-2002, we divided patients into two groups: with low risk (NRS < 3) and with high risk (NRS ≥ 3) of malnutrition. According to SGA, we divided patients into three groups: well-nourished (A), with mild/moderate malnutrition (B), and with severe malnutrition (C). When dichotomous divisions were necessary (for odds ratio—OR or hazard ratio—HR calculations) we considered group A vs. group B or C, as well as group A or B vs. group C. In addition, we assessed whether the patients developed critical weight loss (CWL) during RT. Considering the protocol of observations in our study, we slightly modified the previously proposed definition of CWL (weight loss of >5% from the beginning of RT to Week 4 or >6.25% to Week 7 of therapy) [20]. Furthermore, pretreatment levels of total protein, albumin, prealbumin, and transferrin were determined in peripheral blood.

### 2.4. Bioelectrical Impedance Analysis

Body composition parameters were obtained with the use of bioelectrical impedance analysis (BIA). An ImpediMed bioimpedance analysis SFB7 BioImp device (Pinkenba, QLD, Australia) was used in all BIA measurements. Fat mass (FM) and fat-free mass (FFM) were derived directly from the device. Fat-free mass index (FFMI) and normalized FFMI were calculated using the following formulas: FFMI [kg/m^2^] = FFM [kg]/(height [m])^2^; nFFMI = FFMI + (6.1 × (1.8 − height (m)).

### 2.5. Determination of TNFRSF1A Polymorphism

**Figure 1:**
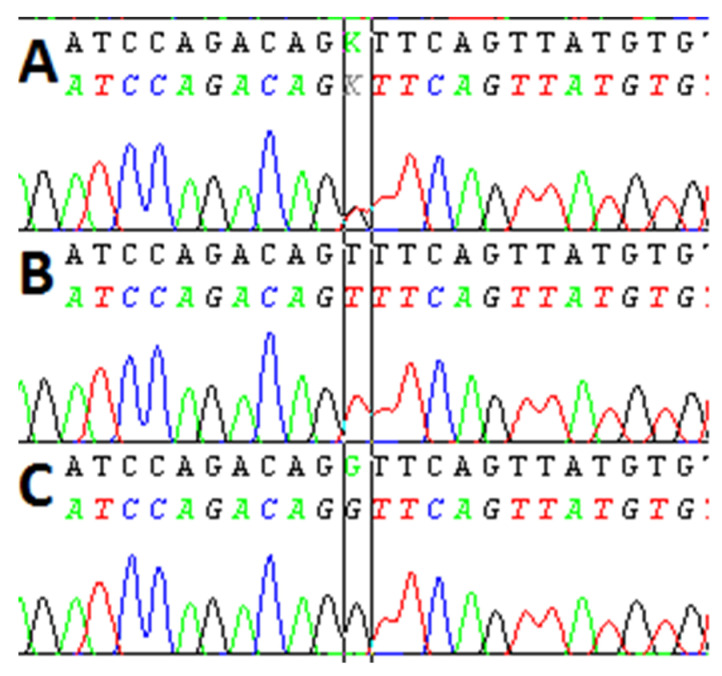
An example of the TNFRSF1A gene sequencing. (**A**–**C**) demonstrate the GT, TT, and GG genotypes of studied SNP (rs4149570), respectively. Specific variants were marked with a black box. Abbreviations: A-green, T-red, C-blue, G-black.

DNA was isolated from peripheral blood using QIAamp DNA Blood Mini Kit (Qiagen, Toronto, ON, Canada). Sequencing was performed using the following primers: forward, 5′ GCC CAC ATC ACT AGC CTT TCC CAG AT 3′ and reverse, 5′ CCA GGA GAC AGG TTA TCT CCA CTC TG 3′, for amplification applying a standard protocol with BigDye Terminator kit v.3.1 cycle sequencing kit (Applied Biosystems, Vilnius, Lithuania) in the Thermoblock 9700. The products of sequencing were purified (EX Terminator- A & A Biotechnology, Gdansk, Poland) and separated by means of capillary electrophoresis using a Genetic Analyzer 3500 (Life Technologies, Carlsbad, CA, USA). The results were analyzed using SeqScape v.3.0 software (Life Technologies, Carlsbad, CA, USA) (Figure 1).

### 2.6. Statistical Analysis

MedCalc version 15.8 (MedCalc Software, Flanders, Belgium) computer software was used for statistical analysis of the obtained data. Categorized data were presented as absolute numbers and percentages. Due to the lack of similar studies on the correlation between SNP of the TNFRSF1A gene and the occurrence of nutritional disorders in HNC patients subjected to IMRT, we decided to use the acquired data to calculate the sample size. SGA status was selected as a primary outcome, while NRS-2002, CWL, parenteral nutrition necessity, and OS were considered secondary outcomes. In the post hoc calculation of sample size, percentages of patients with the TT genotype, as well as with the GT or GG genotype, and primary outcome were used. Most medical studies consider a type I error (alpha) of 0.05 to reject the null hypothesis. In turn, to achieve 80% of statistical power, type II error (beta, 1 − power) was set to 0.20. Considering the percentage of patients with severe malnutrition (C according to SGA) and TT genotype (70%) or with GT or GG genotype (28.36%), and the ratio of sample sizes in compared groups (2.5), it was estimated that the minimal study group should include 66 patients. Moreover, to ensure the appropriate credibility of obtained results, we decided to increase the sample size of minimum 15%. Thus, we estimated that 77 patients should be included in the final analysis. The normality of data distribution was assessed with the D’Agostino–Pearson test. Since continuous data had a non-normal distribution, median and range (in descriptive characteristics) or interquartile range (in comparisons) were used as a measure of their concentration and dispersion, respectively. This was also the reason for the use of a nonparametric test (Mann–Whitney U) in comparisons of continuous variables. Odds ratio (OR) was calculated to assess the risk of nutritional deficiencies (NRS ≥ 3, SGA B or C, SGA C, CWL) according to demographic, clinical, and genetic (TNFRSF1A genotypes) variables. For the purpose of multivariable analysis of the risk of nutritional disorders, logistic regression was applied (all statistically significant variables of univariable analysis were used for adjustment; the only exceptions were T, N or M stage as they were a part of the composite measure—TMN. Similarly, if two or more variants of categorization for a variable were used, only one of them was included in the multivariable analysis; this was applicable in the case of, e.g., tumor location and *TNFRSF1A* genotype.

In univariable survival analysis, a log-rank test with the calculation of the risk coefficient hazard ratio (HR) was used. The Kaplan–Meier estimation method was used for the generation of survival curves. In multivariable survival analysis, Cox logistic regression models were used (adjustment was performed with the same assumptions as for the multivariable analysis of the risk of nutritional disorders). The collinearity of variables included in the multivariable analysis was assessed on the basis of the variance inflation factor (VIF). In all cases, its value was low (<1.2), indicating that no significant collinearity occurred. For the interaction testing, tests of between-subject effects were used. Except for variables being different variants of the same category or components of a composite variable, no significant effects between studied variables were found (*p* > 0.05). In all analyses, two-tailed tests were used, and results with a type I error (alpha) below 5% were considered statistically significant.

## 3. Results

### 3.1. Characteristics of the Study Group

The study included 77 patients with advanced HNC (71.4% of patients were in stage IV according to the TNM classification). The median age was 63 years (range: 42–87 years). Men predominated (80.5%). In 50.6% of patients, the tumor was located in the larynx, whereas, in 37.7%, oropharyngeal cancer was found. All patients received RT treatment (alone or combined with other types of treatment). Parenteral nutrition was used in 19.5% of patients. The characteristics of the study group was presented in Table 1.

### 3.2. The Influence of the Demographic, Clinical, and Genetic Variables on the Risk of Malnutrition According to SGA

Data on the influence of selected demographic, clinical, and genetic variables on the risk of malnutrition according to SGA are presented in Table 2.

#### 3.2.1. Univariable Analysis

Patients with oropharyngeal cancer, compared to the other tumor sites, had a significantly lower risk (fourfold) of moderate or severe malnutrition (62.07% vs. 87.50%; OR = 0.23; *p* = 0.0123). A nearly 21-fold higher risk of moderate or severe malnutrition was noted in patients with T4 stage (97.14% vs. 61.91%; OR = 20.92; *p* = 0.0042). A higher than eightfold risk of moderate or severe malnutrition was found in patients with advanced stage of disease (IV) (89.09% vs. 50%; OR = 8.17; *p* = 0.0005). Moreover, a 24-fold higher risk of moderate or severe malnutrition was found in patients classified as smokers (97.96% vs. 66.67%; OR = 24; *p* = 0.0048).

A nearly threefold higher risk of severe malnutrition was observed in patients with the T4 stage (45.72% vs. 23.81%; OR = 2.69; *p* = 0.0458). An approximately 21-fold higher risk of severe malnutrition was noted in patients with lymph node involvement (N1-N3) (74.08% vs. 12%; OR = 20.95; *p* < 0.0001). Over 40-fold higher risk of severe malnutrition was noted in patients with advanced-stage disease (IV) (47.27% vs. 0%; OR = 40.42; *p* = 0.0110). Nearly fivefold higher risk of severe malnutrition was found in patients classified as smokers (44.90% vs. 14.29%; OR = 4.89; *p* = 0.0095). Moreover, the presence of the TT genotype of the *TNFRSF1A* gene was related to a significantly higher (nearly sixfold) risk of severe malnutrition (70% vs. 28.36%; OR = 5.89; *p* = 0.0167).

#### 3.2.2. Multivariable Analysis

Independent predictors of higher risk of moderate or severe malnutrition included T4 stage (OR = 20.24; *p* = 0.0052) and stage IV disease according to TNM (OR = 9.47; *p* = 0.0011). Interestingly, the location of tumors in the oropharyngeal region was found to be significantly related to a lower risk of moderate or severe malnutrition (OR = 0.18; *p* = 0.0123).

In turn, independent predictors of a higher risk of severe malnutrition included stage IV disease according to TNM (OR = 2.69; *p* = 0.0107), excessive alcohol consumption (OR = 3.99, *p* = 0.0174), and TT genotype of *TNFRSF1A* gene (OR = 5.05, *p* = 0.0350).

### 3.3. The Influence of the Demographic, Clinical, and Genetic Variables on the Nutritional Risk According to NRS-2002

Data on the influence of selected demographic, clinical, and genetic variables on the nutritional risk according to NRS-2002 are presented in Table 3.

#### 3.3.1. Univariable Analysis

None of the studied variables had a significant influence on the nutritional risk (≥3) according to the NRS.

#### 3.3.2. Multivariable Analysis

Since none of the studied variables were revealed to be statistically significant in univariable analysis, the multivariable analysis was abandoned.

### 3.4. The Influence of the Demographic, Clinical, Nutritional, and Genetic Variables on the Risk of the Necessary Application of Parenteral Nutrition

#### 3.4.1. Univariable Analysis

Data on the influence of selected demographic, clinical, nutritional, and genetic variables on the risk of parenteral nutrition necessity are presented in Table 4. Among the assessed variables, only the M1 stage (50% vs. 14.9%; OR = 5.7; *p* = 0.0155), worse PS (>1) (40% vs. 14.5%; OR = 3.93; *p* = 0.0322), and worse SGA status (C) (34.6% vs. 11.8%; OR = 3.97; *p* = 0.0213) were significantly associated with a higher risk of the necessity to apply parenteral nutrition. On the other hand, interestingly, the tumor’s location in the oropharyngeal was significantly associated with a lower risk of the need for parenteral nutrition (6.9% vs. 27.1%; OR = 0.20; *p* = 0.0443) in our study group.

#### 3.4.2. Multivariable Analysis

Location of tumor in oropharyngeal region revealed to be the only significant predictor independently related to lower risk of parenteral nutrition necessity (OR = 0.16; *p* = 0.0439).

### 3.5. The Influence of the Demographic, Clinical, Nutritional, and Genetic Variables on the Risk of CWL

#### 3.5.1. Univariable Analysis

Data on the influence of selected demographic, clinical, nutritional, and genetic variables on the risk of CWL are presented in Table 5. Oropharyngeal cancer was associated with an eightfold higher risk of CWL compared to other tumor sites (65.52% vs. 18.23%; OR = 8.79; *p* = 0.0001). Furthermore, patients with cancer located in the larynx, as compared to other tumor sites, had a more than 10-fold lower risk of CWL (12.82% vs. 60.53%; OR = 0.09; *p* = 0.0001). Moreover, patients with the TT genotype of the *TNFRSF1A* gene had a more than a ninefold higher risk of CWL (80% vs. 29.85%; OR = 9.40; *p* = 0.0072). On the other hand, patients with the GG genotype of the *TNFRSF1A* gene had a nearly fourfold lower risk of CWL (17.39% vs. 44.44%; OR = 0.26; *p* = 0.0298).

#### 3.5.2. Multivariable Analysis

Independent predictors of higher risk of CWL included oropharyngeal location of the tumor (OR = 8.23; *p* = 0.0001) and TT genotype of *TNFRSF1A* gene (OR = 9.40, *p* = 0.0072). Interestingly, the tumor location in the larynx and GG genotype of the *TNFRSF1A* gene were found to be significantly related to a lower risk of CWL (OR = 0.09; *p* = 0.0001, OR = 0.26; *p* = 0.0298, respectively).

### 3.6. Overall Survival

The influence of demographic, clinical, nutritional, and genetic variables on survival is presented in Table 6.

#### 3.6.1. Univariable Analysis

**Figure 2:**
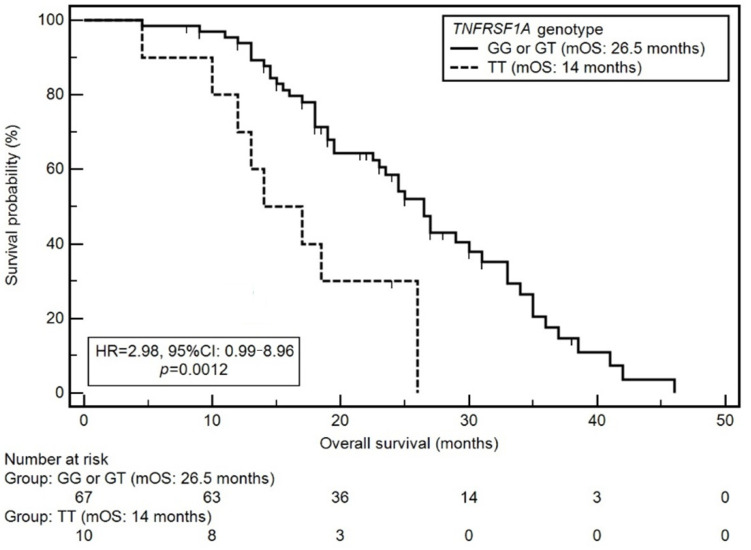
Kaplan–Meier curves showing the probability of overall survival depending on the presence of a specific genotype of the *TNFRSF1A* gene.

The presence of T4 stage (median overall survival—mOS: 23 vs. 30 months; HR = 1.92; *p* = 0.0093), advanced stage of disease (IV) according to the TNM classification (mOS: 24.5 vs. 29 months; HR = 1.89; *p* = 0.0174), the presence of moderate or severe malnutrition (mOS: 23 vs. 35 months; HR = 2.27; *p* = 0.0072), the occurrence of CWL (mOS: 18.5 vs. 27 months; HR = 1.91, *p* = 0.0142), and TT genotype of the *TNFRSF1A* gene (mOS: 14 vs. 26.5 months; HR = 2.98; *p* = 0.0012; Figure 2) were significantly related to a higher risk of death.

#### 3.6.2. Multivariable Analysis

The presence of T4 stage (HR = 2.07; *p* = 0.0193), advanced stage of disease (IV) according to the TNM classification (HR = 2.47; *p* = 0.0203), the occurrence of CWL (HR = 1.92, *p* = 0.0364), and TT genotype of the *TNFRSF1A* gene (HR = 3.02; *p* = 0.0051) were independent, adverse prognostic factors.

### 3.7. Comparisons of Demographic, Laboratory, and Nutritional Variables According to TNFRSF1A Genotypes

Carriers of the TT genotype, compared to patients with the other variants of the *TNFRSF1A* gene, had a significantly lower concentration of prealbumin (median: 15 vs. 0.20 g/dL; *p* = 0.0234). In the case of all other studied variables, no significant differences depending on *TNFRSF1A* genotypes were found (Table S1).

### 3.8. Comparisons of Demographic, Laboratory, and Nutritional Variables According to SGA Category

Well-nourished patients had higher weight (median: 76 vs. 65 kg; *p ≤* 0.0001) and BMI (median: 25.69 vs. 22.44 kg/m^2^; *p ≤* 0.0001) compared to those with moderate or severe malnutrition. Well-nourished patients had a higher albumin concentration compared to patients with moderate or severe malnutrition (median: 3.75 vs. 3.30 g/L; *p* < 0.0001). Patients with moderate or severe malnutrition had a higher value of the nFFMI compared to the well-nourished ones (median: 17.44 vs. 16.38 kg/m^2^; *p* = 0.0297). Well-nourished or moderately malnourished patients had a higher BMI (median: 24.45 vs. 21.55 kg/m^2^; *p* = 0.0126) compared to those with severe malnutrition. Well-nourished or moderately malnourished patients had a higher albumin concentration compared to patients with moderate or severe malnutrition (median: 3.40 vs. 3.22 g/L; *p* = 0.0118). In the case of all other studied variables, no significant differences depending on the SGA category were found (Table S2).

### 3.9. Comparisons of Demographic, Laboratory and Nutritional Variables According to NRS Category

In patients with lower nutritional risk according to the NRS-2002, a higher weight (median: 67.5 vs. 60 kg; *p* = 0.0274) and BMI (median: 22.91 vs. 19.69 kg/m^2^; *p* = 0.0067) were noted. Patients with lower nutritional risk had a significantly higher FFMI (median: 16.78 vs. 16.08 kg/m^2^; *p* = 0.0155) and nFFMI (median: 17.44 vs. 16.38 kg/m^2^; *p* = 0.0129). In the case of all other studied variables, no significant differences depending on the NRS category were found (Table S3).

### 3.10. Comparisons of Demographic, Laboratory and Nutritional Variables According to CWL

Patients without CWL had a significantly higher weight (median: 68.50 vs. 64.00 kg; *p* = 0.0341), FFM (median: 53.47 vs. 45.64 kg; *p* = 0.0026), FFMI (median: 18.07 vs. 16.52 kg/m^2^; *p* = 0.0064), and nFFMI (median: 18.53 vs. 17.15 kg/m^2^; *p* = 0.0134). In the case of all other studied variables, no significant differences depending on CWL occurrence were found (Table S3).

## 4. Discussion

Early identification of patients with the risk of nutritional deficiencies leading to precise and timely implementation of nutritional support can prevent the occurrence of malnutrition or at least the development of its more severe form. Moreover, it may improve patients’ QoL and reduce the risk of death. By identifying novel malnutrition risk factors, we can significantly improve the screening of patients and, thus, apply the necessary nutritional support or modify treatment protocols in patients at risk [21,22]. However, there are still no reliable factors, independent of variables such as disease stage or applied treatment (SNPs, despite the fact that they are not entirely flawless, are not burdened with such disadvantages, as they do not change over the course of the disease and are not treatment-dependent). Moreover, the risk of CRM is individual and seems not to be related to demographic or most of the clinical factors. Therefore, it is suggested that the risk of malnutrition may be related to genetic predispositions [23]. SNPs, because of their stable nature, can be used to predict which patients are at risk of malnutrition and, therefore, who requires early nutritional support [10].

In clinical practice, several tools are used for assessing the nutritional status of patients. One of the most commonly used nutritional screening tests, allowing for the identification of patients at nutritional risk, is NRS-2002 [24]. Other tools facilitating the assessment of the current nutritional status of patients include Patient-Generated Subjective Global Assessment (PG-SGA) or SGA, Malnutrition Universal Screening Tool (MUST), Nutritional Risk Index (NRI), and Malnutrition Screening Tool (MST) [25,26]. In addition to physical examination (occurrence of edema), comprehensive assessment of nutritional status should include anthropometric examination, measurement of laboratory indicators (albumin, transferrin, and lymphocytes), body composition (e.g., using bioelectrical impedance analysis—BIA) or imaging methods (e.g., dual-energy X-ray absorptiometry—DXA). Additionally, in patients with HNC, especially those subjected to (C)RT, the identification of those at risk of critical weight loss (CWL) is of special interest, as its occurrence is associated with poor survival [6]. In particular, CWL related to (C)RT seems to be of high interest to clinicians due to its potential usefulness in the adjustment of the treatment protocols [20,27].

In our study, the presence of the TT genotype of the *TNFRSF1A* gene was related to significantly higher (nearly sixfold) risk of severe malnutrition according to SGA. Other factors significantly related to a higher risk of moderate or severe malnutrition according to SGA included the tumor located in the larynx, T4 stage, lymph node involvement (N1-N3), advanced disease (IV stage according to TNM classification), and smoking. Additionally, carriers of the TT genotype of the studied gene had a significantly higher (over ninefold) risk of CWL. Among other studied factors, only the location of the tumor in the oropharynx was significantly related to a higher risk of CWL. In turn, the location of the tumor in the larynx and GG genotype of the studied gene was significantly related to a lower risk of CWL. In the case of survival, apart from the classic prognostic factors such as T4 stage, advanced stage of disease (IV), and the presence of moderate or severe malnutrition, the occurrence of CWL and the TT genotype of the *TNFRSF1A* gene were significantly related to a higher risk of death. Interestingly, on the basis of multivariate analysis, all these factors except SGA were independent, adverse prognostic factors.

Several studies found that various SNPs of the *TNFRSF1A* gene (including the subject of our research—rs4149570) have functional consequences. Comabella et al. showed that the CT genotype of SNP rs4149584 (located in the coding sequence, R92Q) was significantly related to both increased expression of mRNA for full-length TNF-R1 protein and increased serum sTNF-R level, whereas CC genotype of SNP rs1800693 was significantly related to increased expression of mRNA for Δ6TNF-R1 isoform [28]. Kim et al. found that the presence of the TT genotype of SNP rs4149570 (marked by authors as −329 G/T) was significantly associated with higher expression of mRNA for *TNFRSF1A* [29]. Similarly, Sainz et al. found that the TT genotype of the same SNP rs4149570 (marked by authors as -609 G/T) was significantly associated with higher expression of mRNA for *TNFRSF1A* [19].

It should be noted that, until now, polymorphism −610 T > G (rs4149570) of the *TNFRSF1A* gene was not studied in relation to the risk of nutritional disorders or as a prognostic factor in patients subjected to IMRT due to HNC.

Since no studies regarding the relationship between the studied SNP and nutritional deficiencies have been published yet, we decided to confront our results with those available for other SNPs with emphasis on those located in genes encoding proteins of the TNF–TNF-R axis.

Powrózek et al. examined 62 patients with HNC (at various stages of disease advancement) subjected to RT. They showed that, *TNF-α* polymorphism (rs1799964; −1031 T/C) and plasma level of this cytokine may be related to the occurrence of cachexia (defined on the basis of SGA: patients classified as SGA-B were considered as pre-cachectic, whereas patients classified as SGA-C were considered as cachectic). The authors found that the presence of the CC genotype of *TNF-α* was associated with a higher risk of developing cachexia. Moreover, they showed that patients with the CC genotype of *TNF-α* had higher TNF-α plasma levels. The authors concluded that the presence of the CC genotype of TNF-α could be an objective biomarker of cachexia in HNC patients [30].

Johns et al. studied polymorphisms in various genes associated with cancer cachexia. They recruited 1276 patients with different cancers (at various stages of disease advancement) including esophageal or gastric (*n* = 405), pancreatic (*n* = 158), lung (*n* = 550), and other (*n* = 163). Most patients (98%) were of European descent. Among others, they found that the C allele of *TNF-α* polymorphism (rs1799964) was significantly associated with low skeletal muscle mass (quantification of the muscle mass was defined by CT scans) and weight loss >2% in all examined patients (*p* = 0.010). The authors concluded that proinflammatory cytokines could serve as indicators of muscle wasting in cachexia [31].

On the other hand, de Luis et al. studied 60 patients operated on due to HNC (at various stages of disease advancement). All patients were treated with early enteral nutrition. The authors concluded that polymorphism of the TNF-α (rs1800629; 308G/A) was not associated with levels of inflammatory markers such as prealbumin, transferrin, CRP, TNF-α, IL-6, and the total number of lymphocytes [32].

Our study group was homogeneous in terms of the disease stage (III and IV) and the applied treatment; all patients received RT with the use of the same technique (IMRT). However, it should be kept in mind that HNCs are a heterogeneous group of neoplasms in terms of their location, which influences the differentiated treatment before RT. Therefore, although it should be seen as a limitation of the study, the distribution of demographic and clinical variables of our group reflects the general population of patients with HNC. Other limitations include the small sample size, the fact that we did not perform control for multiple hypothesis testing, and the lack of data regarding the patient eating habits or HPV status. In spite of the limitations, to the best of our knowledge, this is the first study demonstrating that SNP rs4149570 in the *TNFRSF1A* gene may be related to the risk of nutritional deficiencies and survival in patients subjected to IMRT due to HNC.

## 5. Conclusions

The TT genotype (rs4149570) of the *TNFRSF1A* gene may be related to a higher risk of nutritional deficiencies (severe malnutrition and CWL) in patients subjected to the IMRT due to HNC. Moreover, in this group of patients, the TT genotype of the studied gene may serve as an independent, unfavorable prognostic factor.

## Figures and Tables

**Table 1:** Characteristics of the study group.

Variable		Study Group (*n* = 77)
Gender	Male	62 (80.5%)
	Female	15 (19.5%)
Age [years]	Median (range)	63 (42–87)
	≥63	39 (50.6%)
	<63	38 (49.4%)
Histopathological diagnosis	Squamous cell carcinoma	71 (92.2%)
	Non-squamous cell carcinoma	6 (77.8%)
Tumor location	Oropharynx	29 (37.7%)
	Larynx	39 (50.6%)
	Other sites ^a^	9 (11.7%)
T stage	T1	3 (3.9%)
	T2	12 (15.6%)
	T3	27 (35.1%)
	T4	35 (45.4%)
N stage	N0	27 (35.1%)
	N1	9 (11.7%)
	N2	35 (45.4%)
	N3	6 (7.8%)
M stage	M0	67 (87%)
	M1	10 (13%)
Disease stage according to TNM	III	22 (28.6%)
	IVA	40 (51.9%)
	IVB	5 (6.5%)
	IVC	10 (13.0%)
Performance status	≤1	21 (27.3%)
	>1	56 (72.7%)
Type of treatment	Surgery + RT	31 (40.2%)
	Surgery + C-RT	18 (23.4%)
	RT alone	12 (15.6%)
	Induction CTH + RT	3 (3.9%)
	C-RT	8 (10.4%)
	Induction CTH + C-RT	4 (5.2%)
	Induction CTH + Surgery + C-RT	1 (1.3%)
Excessive alcohol consumption	Yes	35 (45.5%)
	No	42 (54.5%)
Smoking status (ever)	Smoker	59 (76.6%)
	Nonsmoker	18 (23.4%)
Smoking status (currently) [no data: *n* = 18]	Current smoker	52 (88.1%)
	Former smoker	7 (11.9%)
Parenteral nutrition	Yes	15 (19.5%)
	No	62 (80.5%)
Weight [kg]	Median (range)	67 (43–91)
BMI [kg/m^2^]	Median (range)	22.83 (14.5–34.4)
SGA	A	17 (22.1%)
	B	34 (44.1%)
	C	26 (33.8%)
NRS-2002	2	54 (70.1%)
	3	20 (26.0%)
	4	2 (2.6%)
	5	1 (1.3%)
CWL	Yes	28 (36.4%)
	No	49 (63.6%)

^a^ Tonsils, jaw, the base of the tongue, and maxillary sinus. Abbreviations: A—well-nourished patients, B—moderately malnourished patients, BMI—body mass index, C—severely malnourished patients, C-RT—chemoradiotherapy, CTH—chemotherapy, CWL—critical weight loss, M—metastatic spread, N—lymph node involvement, NRS-2002—nutritional risk screening 2002, SGA—Subjective Global Assessment, RT—radiotherapy, T—tumor site and size.

**Table 2:** The influence of the demographic, clinical, and genetic variables on the risk of malnutrition according to SGA.

Variable		SGA							
		A	B or C	Univariable Analysis	Multivariable Analysis	A or B	C	Univariable Analysis	Multivariable Analysis
				OR [95% CI] *p*	OR [95% CI] *p*			OR [95% CI] *p*	OR [95% CI] *p*
Gender	Male	11 (55.00%)	51 (89.48%)	3.09 [0.91–10.48] 0.0701	4.67 [0.96–22.63] 0.0555	40 (64.52%)	22 (35.48%)	1.51 [0.43–5.32] 0.5189	1.73 [0.43–6.94] 0.4387
	Female	6 (10.52%)	9 (45.00%)			11 (73.33%)	4 (26.67%)		
Age [years]	≥63	10 (25.64%)	29 (74.36%)	0.65 [0.22–1.95] 0.4467	0.42 [0.11–1.60] 0.2034	25 (64.10%)	14 (35.90%)	1.21 [0.47–3.12] 0.6889	1.20 [0.44–3.33] 0.7216
	<63	7 (18.42%)	31 (81.58%)			26 (68.42%)	12 (31.58%)		
Histopathological diagnosis	Squamous-cell carcinoma	14 (19.72%)	57 (80.28%)	4.07 [0.74–22.37] 0.1063	2.99 [0.35–25.82] 0.3184	46 (64.79%)	25 (35.21%)	2.72 [0.30–24.56] 0.3735	5.02 [0.37–68.27] 0.2252
	Non-squamous-cell carcinoma	3 (50.00%)	3 (50.00%)			5 (83.33%)	1 (16.67%)		
Tumor location	Oropharynx	11 (37.93%)	18 (62.07%)	0.23 [0.07–0.73] 0.0123 *	0.18 [0.05–0.68] 0.0116 *	9 (50.00%)	9 (50.00%)	2.48 [0.83–7.29] 0.1014	0.92 [0.32–2.65] 0.8785
	Other sites ^a^	6 (12.50%)	42 (87.50%)			42 (71.19%)	17 (28.81%)		
	Larynx	5 (12.82%)	34 (87.18%)	3.14 [0.98–10.03] 0.0536	2.72 [0.73–10.09] 0.1352	26 (66.67%)	13 (33.33%)	0.96 [0.37–2.47] 0.9351	0.85 [0.31–2.39] 0.7704
	Other sites ^b^	12 (31.58%)	26 (68.42%)			25 (65.79%)	13 (34.21%)		
T stage	T4	1 (2.86%)	34 (97.14%)	20.92 [2.60–168.12] 0.0042 *	20.24 [2.45–166.82] 0.0052 *	19 (54.28%)	16 (45.72%)	2.69 [1.02–7.13] 0.0458 *	2.23 [0.81–6.20] 0.1221
	T1–3	16 (38.09%)	26 (61.91%)			32 (76.19%)	10 (23.81%)		
N stage	N1–3	8 (16.00%)	42 (84.00%)	2.62 [0.87–7.89] 0.0858	2.90 [0.91–9.29] 0.0722	7 (25.92%)	20 (74.08%)	20.95 [6.23–70.39] <0.0001 *	2.83 [0.88–9.08] 0.0800
	N0	9 (33.33%)	18 (66.67%)			44 (88.00%)	6 (12.00%)		
M stage	M1	1 (10.00%)	9 (90.00%)	2.82 [0.33–24.02] 0.3420	2.78 [0.29–27.12] 0.3766	4 (40.00%)	6 (60.00%)	3.52 [0.89–13.86] 0.0713	4.34 [0.96–19.61] 0.0561
	M0	16 (23.88%)	51 (76.12%)			47 (70.15%)	20 (29.85%)		
Disease stage according to TNM	IVA-IVC	6 (10.91%)	49 (89.09%)	8.17 [2.48–26.86] 0.0005 *	9.47 [2.45–36.56] 0.0011 *	29 (52.73%)	26 (47.27%)	40.42 [2.33–699.65] 0.0110 *	2.69 [1.26–5.74] 0.0107 *
	III	11 (50.00%)	11 (50.00%)			22 (100.00%)	-		
Performance status	>1	12 (19.35%)	50 (80.65%)	2.08 [0.6–7.23] 0.2478	0.42 [0.10–1.85] 0.2550	43 (69.35%)	19 (30.64%)	0.50 [0.16–1.59] 0.2439	2.18 [0.58–8.28] 0.2502
	≤1	5 (33.33%)	10 (66.67%)			8 (53.33%)	7 (46.67%)		
Excessive alcohol consumption	Yes	6 (16.22%)	31 (83.78%)	1.96 [0.64–5.98] 0.2374	1.66 [0.43–6.45] 0.4658	21 (56.76%)	16 (43.24%)	2.69 [1.02–7.13] 0.0458 *	3.99 [1.27–12.52] 0.0174 *
	No	11 (27.50%)	29 (72.50%)			30 (75.00%)	10 (25.00%)		
Smoking status (ever)	Smoker	1 (2.04%)	48 (97.96%)	24.00 [2.63–218.67] 0.0048 *	1.49 [0.35–6.28] 0.5853	27 (55.10%)	22 (44.90%)	4.89 [1.47–16.21] 0.0095 *	2.01 [0.55–7.39] 0.2919
	Nonsmoker	6 (33.33%)	12 (66.67%)			24 (85.71%)	4 (14.29%)		
Smoking status (currently) [no data *n* = 18]	Current smoker	10 (19.23%)	42 (80.77%)	0.70 [0.07–6.49] 0.7535	1.45 [0.12–17.67] 0.7682	33 (63.46%)	19 (36.54%)	0.77 [0.15–3.80] 0.7460	0.69 [0.11–4.31] 0.6939
	Former smoker	1 (14.28%)	6 (85.72%)			4 (57.14%)	3 (42.86%)		
Treatment	Definitive (C)-RT	1 (10.00%)	9 (90.00%)	1.39 [0.43–4.47] 0.5810	2.13 [0.48–9.48] 0.3223	3 (30.00%)	7 (70.00%)	1.60 [0.60–4.26] 0.3432	1.83 [0.57–5.85] 0.3091
	Postoperative (C)-RT	16 (23.88%)	51 (76.12%)			48 (71.64%)	19 (28.36%)		
Concurrent C-RT	Yes	9 (27.27%)	24 (72.73%)	1.04 [0.36–2.97] 0.9459	0.65 [0.17–2.46] 0.5239	21 (63.64%)	12 (36.36%)	1.22 [0.48–3.17] 0.6766	1.32 [0.46–3.83] 0.6052
	No	8 (18.18%)	36 (81.82%)			30 (68.18%)	14 (31.82%)		
*TNFRSF1A* genotype	TT	1 (10.00%)	9 (90.00%)	2.82 [0.33–24.02] 0.3420	0.95 [0.09–9.91] 0.9649	3 (30.00%)	7 (70.00%)	5.89 [1.38–25.21] 0.0167 *	5.05 [1.12–22.76] 0.0350 *
	GT and GG	16 (23.88%)	51 (76.12%)			48 (71.64%)	19 (28.36%)		
	GG	6 (26.09%)	17 (73.91%)	0.72 [0.23–2.27] 0.5808	0.28 [0.06–1.26] 0.0973	16 (69.56%)	7 (30.44%)	0.81 [0.28–2.30] 0.6869	0.72 [0.24–2.13] 0.5495
	TT and GT	11 (20.37%)	43 (79.63%)			35 (64.81%)	19 (35.19%)		
	GT	10 (22.73%)	34 (77.27%)	0.91 [0.31–2.73] 0.8740	3.60 [0.76–17.02] 0.1059	32 (72.73%)	12 (27.27%)	0.51 [0.19–1.32] 0.1668	0.60 [0.22–1.64] 0.3218
	GG and TT	7 (21.21%)	26 (78.79%)			19 (57.58%)	14 (42.42%)		

* Statistically significant results. ^a^ Tonsils, jaw, the base of the tongue, maxillary sinus, and larynx; ^b^ tonsils, jaw, the base of the tongue, maxillary sinus, and oropharynx. Abbreviations: A—well-nourished patients, B—moderate malnutrition, C—severe malnutrition, CI—confidence interval, C-RT—chemoradiotherapy, M—metastatic spread, N—lymph node involvement, OR—odds ratio, SGA—Subjective Global Assessment, T—tumor site and size, *TNFRSF1A*—tumor necrosis factor receptor superfamily member 1A gene.

**Table 3:** The influence of the demographic, clinical, and genetic variables on the nutritional risk according to NRS-2002.

Variable		NRS-2002		
		<3	≥3	OR [95% CI] *p*
Gender	Male	43 (69.35%)	19 (30.64%)	1.21 [0.34–4.31] 0.7628
	Female	11 (73.33%)	4 (26.67%)	
Age [years]	≥63	27 (76.15%)	12 (23.85%)	1.09 [0.41–2.90] 0.8614
	<63	27 (71.05%)	11 (28.95%)	
Histopathological diagnosis	Squamous-cell carcinoma	51 (71.83%)	20 (28.17%)	0.39 [0.07–2.11] 0.2753
	Non-squamous-cell carcinoma	3 (50.00%)	3 (50.00%)	
Tumor location	Oropharyngeal	21 (72.41%)	8 (27.59%)	0.84 [0.30–2.32] 0.7337
	Other ^a^	33 (68.75%)	15 (31.25%)	
	Larynx	26 (66.67%)	13 (33.33%)	1.40 [0.52–3.74] 0.5019
	Other ^b^	28 (73.68%)	10 (26.32%)	
T stage	T4	25 (71.43%)	10 (28.87%)	0.89 [0.33–2.38] 0.8202
	T1–3	29 (69.05%)	13 (30.95%)	
N stage	N1–3	37 (74.00%)	13 (26.00%)	0.60 [0.22–1.63] 0.3147
	N0	17 (62.96%)	10 (37.04%)	
M stage	M1	7 (70.00%)	3 (30.00%)	1.00 [0.24–4.29] 0.9923
	M0	47 (70.15%)	20 (29.85%)	
Disease stage according to TNM	IVA-IVC	41 (74.54%)	14 (25.46%)	0.49 [0.17–1.40] 0.1846
	III	13 (59.09%)	9 (40.91%)	
Performance status	>1	12 (80.00%)	3 (20.00%)	0.52 [0.13–2.07] 0.3575
	≤1	42 (67.74%)	20 (32.26%)	
Excessive alcohol consumption	Yes	25 (71.43%)	10 (28.57%)	0.89 [0.33–2.38] 0.8202
	No	29 (69.05%)	13 (30.95%)	
Smoking status (ever)	Smoker	43 (72.88%)	16 (27.12%)	0.58 [0.19–1.77] 0.3424
	Nonsmoker	11 (61.11%)	7 (38.89%)	
Smoking status (currently) [no data *n* = 18]	Current smoker	37 (71.15%)	15 (28.85%)	2.43 [0.27–21.96] 0.4285
	Former smoker	6 (85.71%)	1 (14.29%)	
Treatment	Definitive (C)-RT	21 (77.8%)	6 (22.2%)	0.55 [0.19–1.63] 0.2846
	Post-operative (C)-RT	33 (66%)	17 (34%)	
Concurrent C-RT	Yes	21 (63.64%)	12 (36.36%)	1.71 [0.64–4.58] 0.2831
	No	33 (75.00%)	11 (25.00%)	
*TNFRSF1A* genotype	TT	10 (100.00%)	-	0.09 [0.01–1.61] 0.1016
	GT and GG	44 (65.67%)	23 (34.33%)	
	GG	14 (60.87%)	9 (39.13%)	1.84 [0.65–5.17] 0.2497
	GT and TT	40 (74.07%)	14 (25.93%)	
	GT	30 (68.18%)	14 (31.82%)	1.24 [0.46–3.36] 0.6665
	GG and TT	24 (72.73%)	9 (27.27%)	

^a^ Tonsils, jaw, the base of the tongue, maxillary sinus, and larynx; ^b^ tonsils, jaw, the base of the tongue, maxillary sinus, and oropharynx. Abbreviations: CI—confidence interval, C-RT—chemoradiotherapy, M—metastatic spread, N—lymph node involvement, NRS-2002—nutritional risk screening 2002, OR—odds ratio, T—tumor site and size, *TNFRSF1A*—tumor necrosis factor receptor superfamily member 1A gene.

**Table 4:** The influence of the demographic, clinical, and genetic variables on the parenteral nutrition necessity.

Variable		*Parenteral Nutrition*			
		No	Yes	Univariable Analysis	Multivariable Analysis
				OR [95% CI] *p*	OR [95% CI] *p*
Gender	Male	52 (83.9%)	10 (16.1%)	0.38 [0.11–1.37] 0.1400	0.44 [0.09–2.18] 0.3172
	Female	10 (66.7%)	5 (33.3%)		
Age [years]	≥63	30 (76.9%)	9 (23.1%)	1.60 [0.51–5.04] 0.4218	1.61 [0.43–6.07] 0.4783
	<63	32 (84.2%)	6 (15.8%)		
Histopathological diagnosis	Squamous-cell carcinoma	58 (81.7%)	13 (18.3%)	0.45 [0.07–2.71] 0.3825	0.24 [0.02–2.65] 0.2457
	Non-squamous-cell carcinoma	4 (66.7%)	2 (33.3%)		
Tumor location	Oropharyngeal	27 (93.1%)	2 (6.9%)	0.20 [0.04–0.96] 0.0443 *	0.16 [0.03–0.95] 0.0439 *
	Other ^a^	35 (72.9%)	13 (27.1%)		
	Larynx	31 (79.5%)	8 (20.5%)	1.14 [0.37–3.54] 0.8168	1.56 [0.40–6.07] 0.5164
	Other ^b^	31 (81.6%)	7 (18.4%)		
T stage	T4	31 (73.8%)	4 (26.2%)	0.36 [0.10–1.27] 0.1121	0.31 [0.08–1.13] 0.0765
	T1–3	31 (88.6%)	11 (11.4%)		
N stage	N1–3	41 (82%)	9 (18%)	0.77 [0.24–2.45] 0.6558	0.51 [0.13–2.03] 0.3390
	N0	21 (77.8%)	6 (22.2%)		
M stage	M1	5 (50%)	5 (50%)	5.70 [1.39–23.35] 0.0155 *	2.66 [0.54–13.01] 0.2267
	M0	57 (85.1%)	10 (14.9%)		
Disease stage according to TNM	IVA-IVC	44 (80%)	11 (20%)	1.12 [0.32–4.00] 0.8556	0.41 [0.06–2.77] 0.3620
	III	18 (81.8%)	4 (18.2)		
Performance status	>1	9 (60%)	6 (40%)	3.93 [1.12–13.72] 0.0322 *	3.68 [0.84–16.08] 0.0827
	≤1	53 (85.5%)	9 (14.5%)		
Excessive alcohol consumption	Yes	25 (71.4%)	10 (28.6%)	2.96 [0.90–9.70] 0.0733	2.58 [0.67–9.90] 0.1653
	No	37 (88.1%)	5 (11.9%)		
Smoking status (ever)	Smoker	47 (79.7%)	12 (20.3%)	1.28 [0.32–5.14] 0.7310	2.35 [0.38–14.48] 0.3574
	Nonsmoker	15 (83.3%)	3 (16.7%)		
Smoking status (currently) [no data *n* = 18]	Current smoker	40 (81.6%)	9 (18.4%)	0.82 [0.26–2.62] 0.7444	0.31 [0.04–2.16] 0.2358
	Former smoker	22 (78.6%)	6 (21.4%)		
Treatment	Definitive (C)-RT	19 (70.4%)	8 (29.6%)	2.59 [0.82–8.16] 0.1051	1.68 [0.43–6.54] 0.4177
	Post-operative (C)-RT	43 (86%)	7 (14%)		
Concurrent C-RT	Yes	9 (69.2%)	4 (30.8%)	2.14 [0.56–8.22] 0.2671	2.04 [0.54–7.70] 0.2943
	No	53 (82.8%)	11 (17.2%)		
NRS-2002	≥3	20 (87%)	3 (13%)	0.52 [0.13–2.07] 0.3575	0.58 [0.13–2.67] 0.4900
	<3	42 (77.8%)	12 (22.2%)		
SGA	B or C	47 (78.3%)	13 (21.7%)	2.07 [0.42–10.26] 0.3708	1.89 [0.27–12.95] 0.5179
	A	15 (88.2%)	2 (11.8%)		
	C	17 (65.4%)	9 (34.6%)	3.97 [1.23–12.84] 0.0213 *	3.40 [0.93–15.52] 0.0650
	A or B	45 (88.2%)	6 (11.8%)		
*TNFRSF1A* genotype	TT	7 (70%)	3 (30%)	1.96 [0.44–8.71] 0.3744	0.71 [0.11–4.52] 0.7165
	GT and GG	55 (82.1%)	12 (17.9%)		
	GG	19 (82.6%)	4 (17.4%)	0.82 [0.23–2.92] 0.7628	0.66 [0.15–2.91] 0.5855
	GT and TT	43 (79.6%)	11 (20.4%)		
	GT	36 (81.8%)	8 (18.2%)	0.82 [0.27–2.56] 0.7399	1.74 [0.43–7.01] 0.4341
	GG and TT	26 (78.8%)	7 (21.2%)		

* Statistically significant results. ^a^ Tonsils, jaw, the base of the tongue, maxillary sinus, and larynx; ^b^ tonsils, jaw, the base of the tongue, maxillary sinus, and oropharynx. Abbreviations: CI—confidence interval, C-RT—chemoradiotherapy, M—metastatic spread, N—lymph node involvement, NRS-2002—nutritional risk screening 2002, OR—odds ratio, SGA—Subjective Global Assessment, T—tumor site and size, *TNFRSF1A*—tumor necrosis factor receptor superfamily member 1A gene.

**Table 5:** The influence of the demographic, clinical, and genetic variables on the critical weight loss risk.

Variable		CWL			
		No	Yes	Univariable Analysis	Multivariable Analysis
				OR [95% CI] *p*	OR [95% CI] *p*
Gender	Male	41 (66.13%)	21 (33.87%)	0.58 [0.19–1.83] 0.3583	0.56 [0.13–2.39] 0.4314
	Female	8 (53.33%)	7 (46.67%)		
Age [years]	≥63	22 (56.41%)	17 (43.59%)	1.89 [0.74–4.88] 0.1841	3.19 [0.90–11.34] 0.0718
	<63	27 (71.05%)	11 (28.95%)		
Histopathological diagnosis	Squamous-cell carcinoma	47 (66.20%)	24 (33.80%)	0.25 [0.04–1.49] 0.1300	0.31 [0.03–3.25] 0.3312
	Non-squamous-cell carcinoma	2 (33.33%)	4 (66.67%)		
Tumor location	Oropharyngeal	10 (34.48%)	19 (65.52%)	8.23 [2.86–23.63] 0.0001 *	15.28 [4.22–55.39] <0.0001 *
	Other ^a^	39 (81.25%)	9 (18.75%)		
	Larynx	34 (87.18%)	5 (12.82%)	0.09 [0.03–0.30] 0.0001 *	0.05 [0.009–0.23] 0.0001 *
	Other ^b^	15 (39.47%)	23 (60.53%)		
T stage	T4	21 (60.00%)	14 (40.00%)	1.81 [0.73–4.51] 0.2028	1.21 [0.36–4.08] 0.7490
	T1–3	38 (73.08%)	14 (26.92%)		
N stage	N1–3	33 (66.00%)	17 (34.00%)	0.75 [0.28–1.94] 0.5579	0.69 [0.20–2.36] 0.5545
	N0	16 (59.26%)	11 (40.74%)		
M stage	M1	7 (100.00%)	3 (30.00%)	0.72 [0.17–3.04] 0.6548	0.80 [0.13–5.00] 0.8097
	M0	42 (62.69%)	25 (37.31%)		
Disease stage according to TNM	IVA-IVC	34 (61.82%)	21 (38.18%)	1.32 [0.46–3.78] 0.6005	0.91 [0.24–3.47] 0.8965
	III	15 (68.19%)	7 (31.82%)		
Performance status	>1	9 (60.00%)	6 (40.00%)	1.21 [0.38–3.85] 0.744	0.62 [0.14–2.82] 0.5396
	≤1	40 (64.52%)	22 (35.48%)		
Excessive alcohol consumption	Yes	23 (65.71%)	12 (34.29%)	0.85 [0.33–2.16] 0.7294	0.90 [0.27–2.96] 0.8645
	No	26 (61.90%)	16 (38.10%)		
Smoking status (ever)	Smoker	35 (59.32%)	24 (40.68%)	2.40 [0.71–8.13] 0.1618	3.83 [0.87–16.85] 0.0759
	Nonsmoker	14 (77.78%)	4 (22.22%)		
Smoking status (currently) [no data *n* = 18]	Current smoker	31 (59.61%)	21 (40.38%)	0.90 [0.18–4.46] 0.9005	0.96 [0.10–9.70] 0.9764
	Former smoker	4 (57.14%)	3 (42.86%)		
Treatment	Definitive (C)-RT	14 (51.9%)	13 (48.1%)	2.17 [0.82–5.70] 0.1172	3.32 [0.89] 0.0731
	Post-operative (C)-RT	35 (70%)	15 (30%)		
Concurrent C-RT	Yes	21 (63.64%)	12 (36.36%)	1.00 [0.39–2.56] 1.0000	0.41 [0.11–1.50] 0.1799
	No	28 (63.64%)	16 (36.36%)		
NRS-2002	≥3	17 (73.91%)	6 (26.09%)	0.51 [0.17–1.51] 0.2252	0.51 [0.13–2.06] 0.3475
	<3	32 (59.26%)	22 (40.74%)		
SGA	B or C	40 (66.67%)	20 (33.33%)	0.56 [0.19–1.68] 0.3023	1.00 [0.25–3.92] 0.9980
	A	9 (52.94%)	8 (47.06%)		
	C	13 (50%)	13 (50%)	2.40 [0.90–6.37] 0.0789	0.98 [0.26–3.62] 0.9725
	A or B	36 (70.59%)	15 (29.41%)		
*Parenteral nutrition*	Yes	10 (66.67%)	5 (33.33%)	0.84 [0.26–2.79] 0.7858	1.54 [0.31–7.76] 0.5995
	No	39 (62.90%)	23 (37.10%)		
*TNFRSF1A* genotype	TT	2 (20.00%)	8 (80.00%)	9.40 [1.83–48.24] 0.0072 *	24.85 [3.74–168.89] 0.0009 *
	GT and GG	47 (70.15%)	20 (29.85%)		
	GG	19 (82.61%)	4 (17.39%)	0.26 [0.08–0.88] 0.0298 *	0.24 [0.6–0.94] 0.0398 *
	GT and TT	30 (55.56%)	24 (44.44%)		
	GT	28 (63.64%)	16 (36.36%)	1.00 [0.39–2.55] 1.0000	0.71 [0.24–2.10] 0.5331
	GG and TT	21 (63.64%)	12 (36.36%)		

* Statistically significant results. ^a^ Tonsils, jaw, the base of the tongue, maxillary sinus, and larynx; ^b^ tonsils, jaw, the base of the tongue, maxillary sinus, and oropharynx. Abbreviations: CI—confidence interval, C-RT—chemoradiotherapy, CWL—critical weight loss, M—metastatic spread, N—lymph node involvement, NRS-2002—nutritional risk screening 2002, OR—odds ratio, SGA—Subjective Global Assessment, T—tumor site and size, *TNFRSF1A*—tumor necrosis factor receptor superfamily member 1A gene.

**Table 6:** The influence of demographic, clinical, nutritional, and genetic variables on survival.

Variable		Univariable Analysis		Multivariable Analysis
		mOS (Months)	HR [95% CI] *p*	HR [95% CI] *p*
Gender	Male	26	0.82 [0.41–1.66] 0.5537	0.58 [0.29–1.17] 0.1335
	Female	23.5		
Age (years)	≥63	25	1.02 [0.60–1.74] 0.9246	0.99 [0.96–1.03] 0.8692
	<63	26.5		
Smoking history (ever)	Yes	24.5	1.36 [0.77–2.41] 0.2777	0.94 [0.47–1.89] 0.8734
	No	26		
Smoking during treatment [no data *n* = 18]	Yes	25	1.32 [0.77–2.25] 0.3006	1.11 [0.60–2.06] 0.7298
	No	26		
Excessive alcohol consumption	Yes	26.5	0.82 [0.48–1.39] 0.4562	0.66 [0.36–1.23] 0.1930
	No	24.5		
Performance status	>1	19.5	1.34 [0.67–2.72] 0.3480	1.63 [0.81–3.30] 0.1742
	≤1	26		
Tumor location	Oropharynx	23.5	0.87 [0.51–1.49] 0.6106	0.83 [0.38–1.82] 0.6489
	Other ^a^	25		
	Larynx	26	0.96 [0.57–1.64] 0.8941	0.85 [0.41–1.76] 0.6671
	Other ^b^	23.5		
T stage	T4	23	1.92 [1.07–3.43] 0.0093 *	2.07 [1.13–3.80] 0.0193 *
	T1-T3	30		
N stage	N1–3	25	1.29 [0.76–2.20] 0.3204	0.85 [0.41–1.78] 0.6692
	N0	26		
M stage	M1	19	1.29 [0.57–2.95] 0.4898	1.04 [0.46–2.32] 0.9251
	M0	25		
Disease stage according to TNM	IVA-IVC	24.5	1.89 [1.11–3.23] 0.0171 *	2.47 [1.16–5.28] 0.0203 *
	III	29		
Parenteral nutrition	Yes	20	1.28 [0.64–2.55] 0.4359	1.23 [0.58–2.61] 0.5939
	No	30.65		
Treatment	Definitive (C)-RT	26.5	0.84 [0.49–1.46] 0.5026	0.84 [0.45–1.58] 0.6000
	Post-operative (C)-RT	19		
Concurrent C-RT	Yes	26.5	0.92 [0.54–1.57] 0.7643	1.25 [0.65–2.37] 0.5033
	No	24.5		
SGA	C	23	1.35 [0.75–2.43] 0.2712	0.72 [0.32–1.60] 0.4194
	A or B	26		
	B or C	23	2.27 [1.31–3.93] 0.0072 *	1.66 [0.66–4.16] 0.2844
	A	35		
NRS-2002	≥3	24.5	0.88 [0.49–1.56] 0.6597	0.77 [0.35–1.68] 0.5165
	<3	26		
CWL	Yes	18.5	1.91 [1.02–3.57] 0.0142 *	1.92 [1.05–3.54] 0.0364 *
	No	27		
*TNFRSF1A* genotype	TT	14	2.98 [0.99–8.96] 0.0012 *	3.02 [1.40–6.50] 0.0051 *
	GG or GT	26.5		
	GG	27	0.67 [0.39–1.17] 0.1750	0.79 [0.36–1.73] 0.5606
	GT or TT	23.5		
	GT	26.5	0.93 [0.54–1.59] 0.7842	1.26 [0.58–2.75] 0.5606
	GG or TT	25		

* Statistically significant result. ^a^ Tonsils, jaw, the base of the tongue, maxillary sinus, and larynx; ^b^ tonsils, jaw, the base of the tongue, maxillary sinus, and oropharynx. Abbreviations: A—well-nourished patients, B—moderately malnourished patients, C—severely malnourished patients, CI—confidence interval, C-RT—chemoradiotherapy, CWL—critical weight loss, HR—hazard ratio, M—metastatic spread, mOS—median overall survival, N—lymph node involvement, N/A—not available, NRS-2002—nutritional risk screening 2002, SGA—Subjective Global Assessment, T—tumor site and size, *TNFRSF1A*—tumor necrosis factor receptor superfamily member 1A gene.

## Data Availability

Not applicable.

## Supplementary Materials

The following supporting information can be downloaded at https://www.mdpi.com/article/10.3390/cancers14143407/s1: Table S1. Comparison of demographic, laboratory, and nutritional variables depending on TNFRSF1A genotypes; Table S2. Comparison of demographic, clinical, and nutritional variables depending on SGA; Table S3. Comparison of demographic, clinical, and nutritional variables depending on NRS or CWL.

## References

[1] Aupérin A. (2020). Epidemiology of head and neck cancers: An update. Curr. Opin. Oncol..

[2] Giraldi L., Leoncini E., Pastorino R., Wünsch-Filho V., de Carvalho M., Lopez R., Cadoni G., Arzani D., Petrelli L., Matsuo K. (2017). Alcohol and cigarette consumption predict mortality in patients with head and neck cancer: A pooled analysis within the International Head and Neck Cancer Epidemiology (INHANCE) Consortium. Ann. Oncol..

[3] Richey L.M., George J.R., Couch M.E., Kanapkey B.K., Yin X., Cannon T., Stewart P.W., Weissler M.C., Shores C.G. (2007). Defining cancer cachexia in head and neck squamous cell carcinoma. Clin. Cancer Res..

[4] Meza-Valderrama D., Marco E., Dávalos-Yerovi V., Muns M.D., Tejero-Sánchez M., Duarte E., Sánchez-Rodríguez D. (2021). Sarcopenia, malnutrition, and cachexia: Adapting definitions and terminology of nutritional disorders in older people with cancer. Nutrients.

[5] Elkashty O.A., Ashry R., Tran S.D. (2019). Head and neck cancer management and cancer stem cells implication. Saudi Dent. J..

[6] Gorenc M., Kozjek N.R., Strojan P. (2015). Malnutrition and cachexia in patients with head and neck cancer treated with (chemo)radiotherapy. Reports Pract. Oncol. Radiother..

[7] Nicolini A., Ferrari P., Masoni M.C., Fini M., Pagani S., Giampietro O., Carpi A. (2013). Malnutrition, anorexia and cachexia in cancer patients: A mini-review on pathogenesis and treatment. Biomed. Pharmacother..

[8] Vanhoutte G., van de Wiel M., Wouters K., Sels M., Bartolomeeussen L., De Keersmaecker S., Verschueren C., De Vroey V., De Wilde A., Smits E. (2016). Cachexia in cancer: What is in the definition?. BMJ Open Gastroenterol..

[9] Baracos V.E. (2018). Cancer-associated malnutrition. Eur. J. Clin. Nutr..

[10] Dianliang Z. (2009). Probing cancer cachexia-anorexia: Recent results with knockout, transgene and polymorphisms. Curr. Opin. Clin. Nutr. Metab. Care.

[11] Yang W., Huang J., Wu H., Wang Y., Du Z., Ling Y., Wang W., Wu Q., Gao W. (2020). Molecular mechanisms of cancer cachexia-induced muscle atrophy (Review). Mol. Med. Rep..

[12] Omatsu H., Kuwahara A., Yamamori M., Fujita M., Okuno T., Miki I., Tamura T., Nishiguchi K., Okamura N., Nakamura T. (2013). TNF-α -857C>T genotype is predictive of clinical response after treatment with definitive 5-fluorouracil/cisplatin-based chemoradiotherapy in Japanese patients with esophageal squamous cell carcinoma. Int. J. Med. Sci..

[13] Sennikov S.V., Vasilyev F.F., Lopatnikova J.A., Shkaruba N.S., Silkov A.N. (2014). Polymorphisms in the tumor necrosis factor receptor genes affect the expression levels of membrane-bound type I and type II receptors. Mediators Inflamm..

[14] Kalliolias G.D., Ivashkiv L.B. (2016). TNF biology, pathogenic mechanisms and emerging therapeutic strategies. Nat. Rev. Rheumatol..

[15] Brenner D., Blaser H., Mak T.W. (2015). Regulation of tumour necrosis factor signalling: Live or let die. Nat. Rev. Immunol..

[16] Sode J., Bank S., Vogel U., Andersen P.S., Sørensen S.B., Bojesen A.B., Andersen M.R., Brandslund I., Dessau R.B., Hoffmann H.J. (2018). Genetically determined high activities of the TNF-alpha, IL23/IL17, and NFkB pathways were associated with increased risk of ankylosing spondylitis. BMC Med. Genet..

[17] Bank S., Skytt Andersen P., Burisch J., Pedersen N., Roug S., Galsgaard J., Ydegaard Turino S., Broder Brodersen J., Rashid S., Kaiser Rasmussen B. (2014). Polymorphisms in the inflammatory pathway genes TLR2, TLR4, TLR9, LY96, NFKBIA, NFKB1, TNFA, TNFRSF1A, IL6R, IL10, IL23R, PTPN22, and PPARG are associated with susceptibility of inflammatory bowel disease in a Danish cohort. PLoS ONE.

[18] Brzozowska A., Powrózek T., Homa-Mlak I., Mlak R., Ciesielka M., Gołębiowski P., Małecka-Massalska T. (2018). Polymorphism of Promoter Region of TNFRSF1A Gene (−610 T > G) as a Novel Predictive Factor for Radiotherapy Induced Oral Mucositis in HNC Patients. Pathol. Oncol. Res..

[19] Sainz J., Salas-Alvarado I., López-Fernández E., Olmedo C., Comino A., García F., Blanco A., Gómez-Lopera S., Oyonarte S., Bueno P. (2010). TNFR1 mRNA expression level and TNFR1 gene polymorphisms are predictive markers for susceptibility to develop invasive pulmonary aspergillosis. Int. J. Immunopathol. Pharmacol..

[20] Langius J.A.E., Bakker S., Rietveld D.H.F., Kruizenga H.M., Langendijk J.A., Weijs P.J.M., Leemans C.R. (2013). Critical weight loss is a major prognostic indicator for disease-specific survival in patients with head and neck cancer receiving radiotherapy. Br. J. Cancer.

[21] Meriggi F. (2015). Cancer Cachexia: One Step Ahead. Rev. Recent Clin. Trials.

[22] Cao J., Xu H., Li W., Guo Z., Lin Y., Shi Y., Hu W., Ba Y., Li S., Li Z. (2021). Nutritional assessment and risk factors associated to malnutrition in patients with esophageal cancer. Curr. Probl. Cancer.

[23] Johns N., Tan B.H., MacMillan M., Solheim T.S., Ross J.A., Baracos V.E., Damaraju S., Fearon K.C.H. (2014). Genetic basis of interindividual susceptibility to cancer cachexia: Selection of potential candidate gene polymorphisms for association studies. J. Genet..

[24] Ostrowska J., Sulz I., Tarantino S., Hiesmayr M., Szostak-Węgierek D. (2021). Hospital Malnutrition, Nutritional Risk Factors, and Elements of Nutritional Care in Europe: Comparison of Polish Results with All European Countries Participating in the nDay Survey. Nutrients.

[25] Zhang Z., Wan Z., Zhu Y., Zhang L., Zhang L., Wan H. (2021). Prevalence of malnutrition comparing NRS2002, MUST, and PG-SGA with the GLIM criteria in adults with cancer: A multi-center study. Nutrition.

[26] Barbosa-Silva M.C.G., Barros A.J.D. (2006). Indications and limitations of the use of subjective global assessment in clinical practice: An update. Curr. Opin. Clin. Nutr. Metab. Care.

[27] Jager-Wittenaar H., Dijkstra P.U., Vissink A., Van Der Laan B.F.A.M., Van Oort R.P., Roodenburg J.L.N. (2007). Critical weight loss in head and neck cancer—Prevalence and risk factors at diagnosis: An explorative study. Support. Care Cancer.

[28] Comabella M., Caminero A.B., Malhotra S., Agulló L., Fernández O., Reverter F., Vandenbroeck K., Rodríguez-Antigüedad A., Matesanz F., Izquierdo G. (2013). TNFRSF1A polymorphisms rs1800693 and rs4149584 in patients with multiple sclerosis. Neurology.

[29] Kim S., Moon S.-M., Kim Y.S., Kim J.-J., Ryu H.-J., Kim Y.-J., Choi J.-W., Park H.-S., Kim D.-G., Shin H.-D. (2008). TNFR1 promoter -329G/T polymorphism results in allele-specific repression of TNFR1 expression. Biochem. Biophys. Res. Commun..

[30] Powrózek T., Mlak R., Brzozowska A., Mazurek M., Gołębiowski P., Małecka-Massalska T. (2018). Relationship between TNF-α −1031T/C gene polymorphism, plasma level of TNF-α, and risk of cachexia in head and neck cancer patients. J. Cancer Res. Clin. Oncol..

[31] Johns N., Stretch C., Tan B.H.L., Solheim T.S., Sørhaug S., Stephens N.A., Gioulbasanis I., Skipworth R.J.E., Deans D.A.C., Vigano A. (2017). New genetic signatures associated with cancer cachexia as defined by low skeletal muscle index and weight loss. J. Cachexia. Sarcopenia Muscle.

[32] de Luis D.A., Sagrado M.G., Vallejo L.A., Carcedo L.M.G., Izaola O., Cuellar L., Terroba M.C., Aller R. (2007). Influence of G308A polymorphism of tumor necrosis factor-alpha gene on inflammatory markers in postsurgical head and neck cancer patients with early enteral nutrition. Nutrition.

