# Supplementary material for: TNFRSF1A Gene Polymorphism (−610 T > G, rs4149570) as a Predictor of Malnutrition and a Prognostic Factor in Patients Subjected to Intensity-Modulated Radiation Therapy Due to Head and Neck Cancer
Source: Cancers (Basel). 2022 Jul 13;14(14):3407. doi: 10.3390/cancers14143407 (PMC9317796; doi:10.3390/cancers14143407)
Supplement: Supplementary file 1 [file cancers-14-03407-s001.zip › cancers-1789343-supplementary.pdf]

Supplementary Materials

TNFRSF1A Gene Polymorphism (−610T > G, rs4149570) as a Predictor of Malnutrition and a Prognostic Factor in Patients Subjected to Intensity-Modulated Radiation Therapy Due to Head and Neck Cancer

Table S1. Comparison of demographic, laboratory and nutritional variables depending on *TNFRSF1A* genotypes.

Variable	***TNFRSF1A* genotype median (interquartile range)**					
	**TT**	**GT and GG**	***p***	**GG**	**GT and TT**	***p***
Age [years]	61.50 (51.00-70.00)	63.00 (56.25-68.75)	0.5696	65.00 (58.50-71.00)	61.00 (56.00-67.00)	0.1189
Weight [kg]	69.00 (67.00-78.00)	66.00 (55.00-71.75)	0.0866	68.00 (58.50-79.50)	66.00 (55.0-70.00)	0.0785
BMI [kg/m^2]	22.95 (22.84-26.73)	22.84 (19.70-25.04)	0.2752	24.54 (20.18-26.86)	22.84 (19.72-24.69)	0.1575
Total protein [g/L]	6.43 (6.16-6.71)	6.64 (6.22-7.02)	0.2686	6.78 (6.03-7.07)	6.58 (6.22-6.88)	0.7051
Albumin [g/L]	3.40 (3.11-3.75)	3.37 (3.22-3.55)	0.9939	3.31 (3.17-3.53)	3.38 (3.24-3.56)	0.2727
Prealbumin[g/dL]	0.20 (0.10-0.20)	0.20 (0.20-0.30)	0.0234*	0.20 (0.20-0.30)	0.20 (0.20-0.20)	0.7921
Transferrin [g/L]	2.50 (2.30-2.70)	2.30 (1.92-3.07)	0.7557	2.10 (1.52-3.17)	2.50 (2.10-2.90)	0.2896
FM [kg]	20.47 (14.40-24.89)	18.56 (13.62-23.63)	0.6011	19.95 (11.88-24.72)	18.48 (13.88-23.49)	0.9026
FM%	26.94 (23.67-34.52)	26.70 (22.53-34.59)	0.9215	26.94 (22.08-36.69)	26.70 (23.65-33.82)	0.7638
FFM [kg]	50.70 (44.35-57.27)	47.58 (44.59-53.07)	0.3592	47.57 (44.63-52.60)	47.75 (44.52-53.68)	0.6009
FFM%	72.90 (65.48-76.54)	73.94 (65.32-77.48)	0.8026	73.24 (63.35-81.25)	73.94 (66.04-76.54)	0.7980
FFMI [kg/m^2]	17.70 (16.59-18.28)	16.53 (14.97-18.43)	0.1899	16.50 (16.00-16.79)	16.75 (14.85-18.46)	0.3614
nFFMI [kg/m^2]	18.50 (17.20-18.56)	17.22 (15.27-18.75)	0.2283	16.98 (16.38-17.71)	17.41 (15.16-18.81)	0.4101

* - statistically significant result.

Abbreviations: BMI - body mass index, FM – fat mass, FFM – fat-free mass, FFMI - fat-free mass index, nFFMI - normalized fat-free mass index, *TNFRSF1A* - Tumor Necrosis Factor Receptor Superfamily Member 1A gene.

Table S2. Comparison of demographic, clinical and nutritional variables depending on SGA.

**Variable**	**SGA** median (interquartile range)					
	**A**	**B and C**	***p***	**A and B**	**C**	***p***
Age [years]	65.00 (53.75-71.50)	61.50 (57.00-68.00)	0.7587	61.00 (56.00-67.00)	64.50 (58.00-71.00)	0.3856
Weight [kg]	76.00 (69.75-83.00)	65.00 (54.50-68.00)	<0.0001*	68.00 (57.25-76.00)	66.00 (55.00-68.00)	0.2669
BMI [kg/m^2]	25.69 (24.53-30.12)	22.44 (19.31-23.60)	<0.0001*	24.45 (19.86-25.71)	21.55 (18.96-22.91)	0.0126*
Total protein[g/L]	6.69 (6.48-7.05)	6.58 (6.21-6.95)	0.4036	6.61 (6.24-6.95)	6.60 (6.16-7.03)	0.6165
Albumin [g/L]	3.75 (3.44-3.93)	3.30 (3.19-3.46)	<0.0001*	3.40 (3.27-3.65)	3.22 (3.14-3.45)	0.0118*
Prealbumin[g/dL]	0.20 (0.20-0.20)	0.20 (0.20-0.20)	0.6641	0.20 (0.20-0.27)	0.20 (0.20-0.30)	0.6329
Transferrin [g/L]	2.50 (1.80-2.08)	2.50 (2.00-2.95)	0.7678	2.30 (2.00-2.97)	2.60 (2.10-2.90)	0.7340
FM [kg]	19.96 (15.79-23.31)	17.24 (12.36-24.99)	0.3022	18.65 (13.88-23.63)	20.34 (11.88-30.17)	0.9313
FM%	25.24 (24.30-33.82)	27.54 (22.20-35.40)	0.9413	26.70 (23.66-34.06)	29.36 (20.18-34.61)	0.9570
FFM [kg]	45.55 (41.11-54.22)	47.85 (44.67-53.22)	0.8538	45.86 (44.52-53.18)	50.65 (44.98-53.47)	0.2719
FFM%	74.76 (66.04-75.88)	72.58 (64.62-79.08)	0.9902	73.94 (66.04-76.54)	70.63 (65.15-80.44)	0.9399
FFMI [kg/m^2]	16.38 (14.28-16.73)	16.75 (15.82-18.46)	0.0654	16.53 (14.65-18.27)	16.80 (15.66-18.46)	0.2154
nFFMI [kg/m^2]	16.38 (15.02-17.38)	17.44 (16.15-19.01)	0.0297*	17.22 (15.08-18.55)	17.56 (15.93-19.01)	0.2276

* - statistically significant result.

Abbreviations: A – well-nourished patients, B – moderately malnourished patients, BMI - body mass index, C – severely malnourished patients, FM – fat mass, FFM – fat-free mass, FFMI - fat-free mass index, nFFMI - normalized fat-free mass index, SGA – Subjective Global Assessment.

Table S3. Comparison of demographic, clinical and nutritional variables depending on NRS or CWL.

Variable	**NRS-2002** median (interquartile range)			**CWL** median (interquartile range)		
	**<3**	**≥3**	***p***	**No**	**Yes**	***p***
Age [years]	62.00 (56.00-70.00)	63.00 (56.50-66.00)	0.9911	64.00 (57.00-68.00)	61.00 (56.00-70.00)	0.9114
Weight [kg]	67.50 (58.00-74.00)	60.00 (51.25-67.75)	0.0274*	68.50 (65.00-75.00)	64.00 (54.75-69.00)	0.0341*
BMI [kg/m^2]	22.91 (21.09-25.69)	19.69 (18.29-24.20)	0.0067*	22.90 (21.48-25.37)	22.84 (19.69-25.10)	0.4649
Total protein[g/L]	6.60 (6.21-6.93)	6.61 (6.22-7.09)	0.7894	6.70 (6.24-7.09)	6.55 (6.22-6.88)	0.2825
Albumin [g/L]	3.34 (3.19-3.52)	3.41 (3.26-3.58)	0.5156	3.37 (3.19-3.69)	3.36 (3.23-3.55)	0.9785
Prealbumin[g/dL]	0.20 (0.20-0.30)	0.20 (0.20-0.27)	0.4920	0.20 (0.20-0.20)	0.20 (0.20-0.30)	0.8167
Transferrin [g/L]	2.50 (2.00-3.10)	2.20 (1.82-2.67)	0.1721	2.25 (2.00-2.70)	2.50 (1.97-3.10)	0.4171
FM [kg]	19.95 (14.40-29.96)	16.17 (11.88-23.09)	0.0508	19.63 (15.26-27.72)	16.79 (11.71-23.62)	0.1340
FM%	26.94 (23.65-34.52)	26.29 (21.97-34.49)	0.6126	26.09 (23.90-33.94)	26.70 (21.97-35.07)	0.7387
FFM [kg]	40.06 (44.98-53.47)	45.86 (43.25-52.60)	0.1495	53.47 (45.75-57.35)	45.64 (44.52-50.46)	0.0026*
FFM%	73.24 (65.48-76.54)	73.94 (65.45-82.48)	0.3945	74.00 (66.06-76.20)	73.94 (64.89-82.48)	0.6603
FFMI [kg/m^2]	16.78 (16.14-18.54)	16.08 (14.50-16.74)	0.0155*	18.07 (16.26-19.03)	16.52 (14.74-16.80)	0.0064*
nFFMI [kg/m^2]	17.44 (16.63-18.81)	16.38 (14.74-17.63)	0.0129*	18.53 (16.51-20.02)	17.15 (15.07-17.79)	0.0134*

* - statistically significant result.

Abbreviations: BMI - body mass index, CWL – critical weight loss, FM – fat mass, FFM – fat-free mass, FFMI - fat-free mass index, nFFMI - normalized fat-free mass index, NRS-2002 – nutritional risk screenng2002.
